# Exploring transferability of plastic-water hyacinth interaction and detection in rivers

**DOI:** 10.1016/j.isci.2026.116238

**Published:** 2026-06-04

**Authors:** Giel W.A. Hagenbeek, Tim H.M. van Emmerik, Tianlong Jia, Pummarin Khamdahsag, Kittiphon Boonma, Riccardo Taormina, Thomas Mani, Marc Rußwurm

**Affiliations:** 1 Geo-information Science and Remote Sensing Laboratory, Wageningen University and Research, Droevendaalsesteeg 3 6708 PB Wageningen Gelderland, the Netherlands; 2 Hydrology and Environmental Hydraulics Group, Wageningen University and Research, Droevendaalsesteeg 3 6708 PB Wageningen Gelderland, the Netherlands; 3 Delft University of Technology, Faculty of Civil Engineering and Geosciences, Department of Water Management, Stevinweg 1, 2628 CN Delft, the Netherlands; 4 Karlsruhe Institute of Technology (KIT), Institute of Water and Environment, Karlsruhe, Germany; 5 Sustainable Environment Research Institute, Chulalongkorn University, Bangkok 10330, Thailand; 6 Geoinformatics Center, Asian Institute of Technology, Pathumthani, Thailand; 7 The Ocean Cleanup, Rotterdam, the Netherlands; 8 University of Bonn, Institute for Food and Resource Economics, Bonn, Germany

**Keywords:** Environmental monitoring, Environmental science, Environmental technology

## Abstract

Rivers are major pathways for plastic pollution to oceans, with high emissions in tropical regions. Research in the Saigon River showed that invasive water hyacinths (WHs) can trap macroplastics and serve as proxies for detecting river plastic using remote sensing. We explore this phenomenon and its detection methods transferability to the Chao Phraya River. Along a 62.1 km river course, WHs trapped an average of 32% of floating plastics, reaching local maxima of 78%, comparable to 54%–82% in the Saigon. Plastic concentration in WHs was 59 times higher than in open water, increasing downstream. Object detection models transferred well for WHs and entangled plastics (Chao Phraya: mAP_50_ = 68% and 54%; Saigon River: mAP_50_ = 70% and 52%) but poorly for free-floating plastics (23% vs. 48%). Physical sampling found 14 times more plastics within WHs than imagery, highlighting WHs’ role in trapping plastics and their potential for monitoring and targeted clean-up efforts.

## Highlights

• Water hyacinths trap up to 78% of floating plastics in the Chao Phraya River
• Hyacinth trapping ratio decreases downstream, while plastic per hyacinth increases
• Physical sampling found 14× more plastics within hyacinths than imagery
• Automated free-floating plastic detection needs local refinement for transferability

## Graphical abstract

**Figure:**
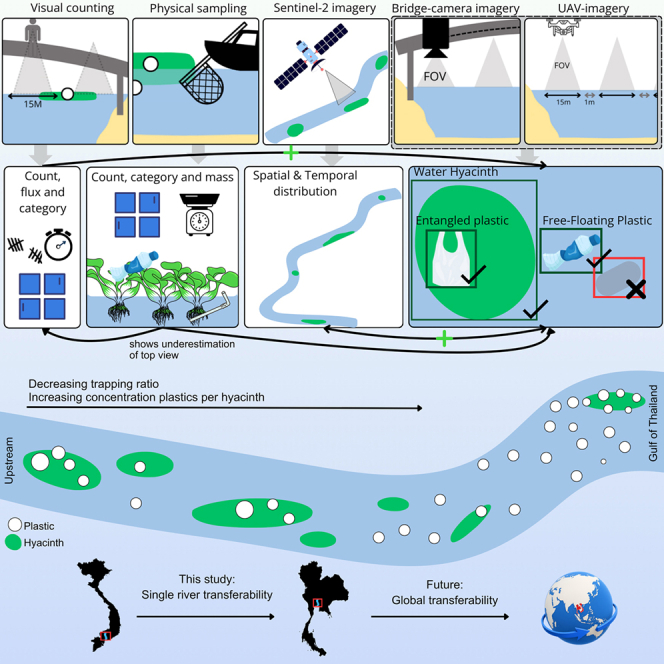

## Introduction

Marine, freshwater, and terrestrial environments are increasingly polluted with plastic waste. This widespread pollution threatens ecosystems, biodiversity, human health, livelihoods, and economic productivity.^1^ Rivers play a significant role in transporting land-based plastics toward the oceans, with annual river plastic emissions estimated to range from 0.8 to 2.7 million tons.^2^ To support plastic pollution reduction and prevention strategies, monitoring of plastic pollution is crucial.^3^^,^^4^^,^^5^ The United Nations Environment Program (UNEP), in its draft legally binding instrument on plastic pollution, emphasizes the importance of identifying plastic hotspots in developing reduction strategies.^6^ Satellite remote sensing (RS) is a relevant method for upscaling measurements and has shown the potential to monitor macroplastics in marine environments.^7^^,^^8^^,^^9^^,^^10^^,^^11^ However, the spatial resolution of RS imagery is often too low for plastic debris detection, limiting its use to large plastic patches and items, leading to an underestimation of total plastic debris.^12^^,^^13^^,^^14^^,^^15^^,^^16^ Therefore, in-situation data collection is required for plastic detection, making coverage of large areas difficult due to the labor, costs, and time involved.^17^ To successfully monitor riverine macroplastics from space, debris patterns, such as vegetation and wood, are being explored as detectable indicators.^12^^,^^18^

Tropical and subtropical rivers dominate global riverine plastic emissions.^2^^,^^19^^,^^20^ In addition, tropical rivers are increasingly infested with water hyacinths (WHs), *Pontederia crassipes* (formerly *Eichhornia crassipes*). This free-floating weed, with fast growth, reproducibility, and light-blocking mat-like clustering, is regarded as one of the most invasive aquatic weeds worldwide, and ways to monitor and eradicate them are actively sought.^21^^,^^22^ At the same time, WHs have been shown to trap or entangle debris, such as plastics. Research in the Saigon River, Vietnam found that of all floating plastics, between 54% and 82% at specific cross-sections, and up to 73% over a 42 km spatial extent were trapped in WHs.^23^^,^^24^^,^^25^ RS using Sentinel-2 satellite imagery has shown to be successful in detecting WH patches in freshwater bodies, including tropical rivers.^26^^,^^27^ Given that (1) WHs can be detected with RS and (2) WHs act as a plastic-trapping mechanism, WH coverage can be used as a proxy for detecting plastics from space.^24^^,^^28^^,^^29^

Beyond the Saigon River, anecdotal evidence of the co-occurrence of plastics and WHs has been reported in other tropical rivers like the Ozama River in the Dominican Republic, the Citarum in Indonesia, the Vam Co Dong River in Vietnam and Chao Phraya River in Thailand.^12^^,^^30^^,^^31^ To use WHs as a generally applicable proxy indicator for macroplastics in rivers, its transferability to other river systems needs to be assessed.^24^ The found relationship between plastics and WHs over the Saigon River’s spatial extent supports the potential transferability of this phenomenon to other hyacinth-invaded fluvial systems.^24^^,^^25^ However, the drivers of plastic transport dynamics in rivers, especially in complex areas such as confluences and tidal regimes, remain poorly understood, creating uncertainties in plastic transport estimates and scaling efforts.^31^^,^^32^ Although the primary factors driving macroplastic transport are not fully understood, WHs can facilitate this transport either through their trapping mechanisms, by exhibiting dynamics similar to those of plastics, or simply by coinciding spatially with them.^24^ Understanding these interactions in different fluvial systems is therefore essential when using WHs as a proxy for plastic pollution.

Previous assessments used two main models to investigate plastic-hyacinth interactions. First, a Sentinel-2 approach using a Naive-Bayes classifier was used to detect WHs in the Saigon River. While this automated classification approach is assumed to be transferable to other river systems, the classification’s true applicability remains uncertain, particularly due to challenges such as cloud cover, WH patch sizes, and seasonal variability.^26^ Second, an object detection deep learning model using You Only Look Once v8 (YOLOv8),^33^ detecting plastics and WHs on *in-situ* UAV (uncrewed aerial vehicle) and fixed camera imagery has been developed on data of the Saigon River.^25^ The application of this approach to another river system remains unknown. This reflects a broader knowledge gap regarding the generalization performance of deep learning models for detecting macroplastic litter across varying geographical and environmental conditions.^34^ This paper has three specific goals. First, we aim to evaluate how Sentinel-2 WH detection is applicable as a valid and useful method when applied to a different river system. Second, we aim to define detection model transferability for plastics and WHs in on-site imagery. Lastly, we aim to determine whether plastic-hyacinth interactions are specific for the Saigon, or whether this phenomenon can serve as a reliable proxy for riverine macroplastic pollution in a different geographic and hydrological context, the Chao Phraya River in this case. With this paper, we assess the transferability of plastic-WH interactions, and field-based and RS detection methods.

## Results

### Study area and methods summary

The Chao Phraya River is approximately 372 km long and flows from northern Thailand through Bangkok before discharging into the Gulf of Thailand.^35^ With its basin of approximately 158,000 km^2^, it is the fifth largest basin in Southeast Asia. The basin accounts for 30% of Thailand’s surface area, making it one of the most important resources for irrigation, transportation, and fishery of the country.^36^ Within the Bangkok Metropolitan Area, discharge averages around 700 m^3^s^−1^, and peaks up to 6,000 m^3^s^−1^.^37^ The river’s discharge is highly influenced by the tropical monsoon climate, resulting in three distinct seasons: hot (February-May), rainy (May-October), and dry (October-February). The lower Chao Phraya discharge is further influenced by tides, leading to fluctuations in discharge of up to ±3,000 m^3^s^−1^. Tidal ranges can exceed 3 m and tidal intrusion reaches up to 175 km upstream during low flow periods and up to 75 km during high stream flow periods.^38^^,^^39^

**Figure 1:**
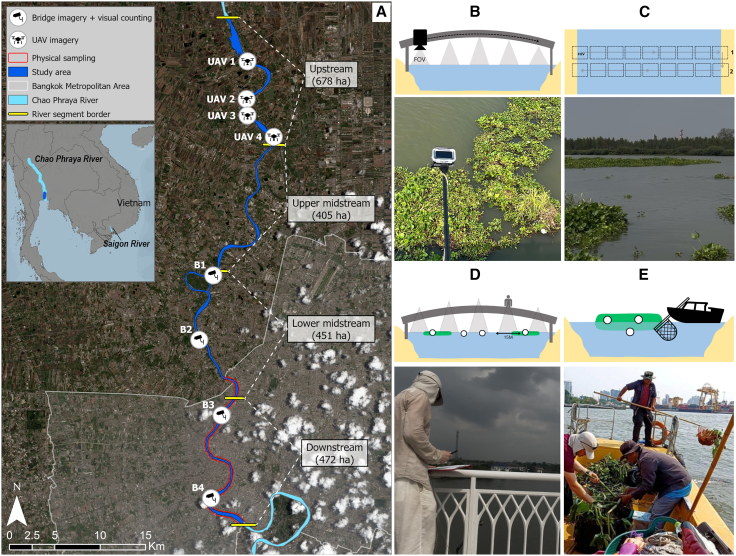
Overview of the study area and in-field data collection methods (A) Study area and location of *in situ* data collection locations, with an RGB (red, green, and blue) Sentinel-2 scene from April 25, 2025 as basemap. (B–E) Schematic representations and in-field photos of the methods bridge-mounted camera imagery, UAV-imagery, visual counting, and physical sampling, respectively.

Sentinel-2 imagery was used to retrieve WH coverage of the entire study area (Figure 1). We collected *in situ* imagery using UAVs and bridge-mounted cameras, over a 62.1 km river extent. Imagery was used as input for object detection of plastics and WHs, retrieving their interaction metrics (Table S3) and allowing for comparison with satellite-derived WH estimates. Visual plastic counting and physical sampling was performed to validate plastic density and support the interpretation of object detection results. Full methodology for each of the imagery processing and field-data collection methods are in STAR Methods.

### Object detection is transferable for hyacinths and entangled plastics

The YOLOv8 Hyacinth Model trained on Saigon River shows only a small 2% mAP50 drop when deployed on Chao Phraya, decreasing from 70% to 68%. The Plastic Model accuracy differs per class. While entangled plastics detection has 54% mAP50 (vs. 52% in Saigon), free-floating plastics detection performs notably worse in Chao Phraya at 23% mAP50 compared to 48% in Saigon. While the classes hyacinths and entangled plastics transfer relatively well, the transferability of detecting free-floating plastics is limited (Table 1). The poor performance could be attributed to site-specific characteristics of the water surface, such as sediment levels, water color, flow velocity, wave patterns, sunglint and the dominant plastic items per region. The composition of plastics, and consequently the distribution of plastic sizes, differs greatly per river and within rivers,^29^^,^^31^ possibly underestimating certain plastic types that are prevalent in different regions. These variable circumstances suggest that free-floating plastic detection could benefit from a highly diverse and complete training set for global transferability, or river-specific calibration for a targeted river. Figure S2 further shows the distribution of detected size classes and occurrence and relationship with sunglint, also highlighting the importance for future work on detection performance and site-specific characteristics.

**Table 1:** Model performance evaluation shows only a small accuracy when evaluating the Saigon-trained YOLO model on the Chao Phraya River

Model	Class	Chao Phraya River (this study)				Saigon River (comparison)^25^			
		precision	recall	(m)AP50	(m)AP50–95	precision	recall	(m)AP50	(m)AP50–95
Hyacinth Model	water hyacinth	0.80	0.54	68%	49%	0.83	0.54	70%	48%
Plastic Model	free-floating plastics	0.29	0.19	23%	16%	0.72	0.25	48%	37%
	entangled plastics	0.52	0.31	54%	35%	0.69	0.39	52%	38%

A default confidence threshold of 0.5 is used for model performance evaluation.

**Table 2:** Plastic concentration entangled in water hyacinths

Measuring technique	Plastic items per m^2^	Mass concentration (g/kg)
Physical sampling	73.91	25.06
Bridge imagery (B3 + B4)	5.33	N/A

**Figure 2:**
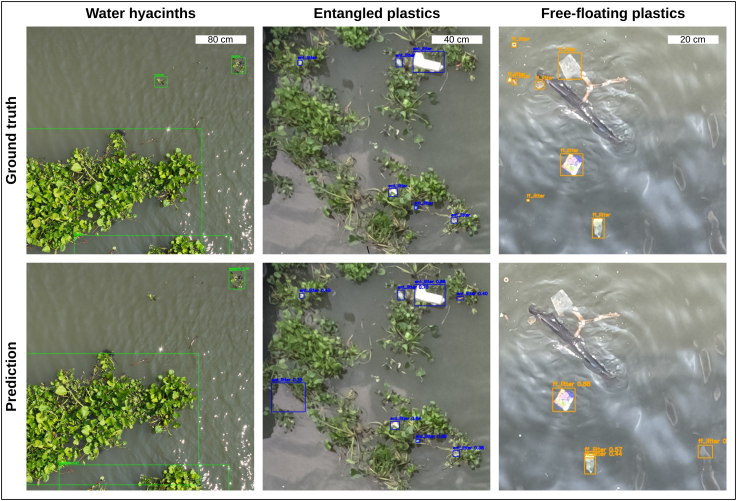
Examples of hand-annotated ground-truth bounding boxes (top) compared to predicted bounding boxes (bottom) for water hyacinths, entangled plastics, and free-floating plastics (left to right) Scale bars represent 80 cm, 40 cm, and 20 cm, respectively.

The good performance of the Hyacinth Model can be attributed to the consistent size and color characteristics of WHs that make their detection well transferable across river systems. Similarly, these characteristics of WHs serve as a distinctive background for entangled plastics,^24^ which may enhance the transferability of their detection, especially compared to free-floating litter against the variable water surface background. Under sunglint conditions, the distinction between sunglint on the water surface and white or transparent free-floating litter items becomes less clear. As a result, the model often fails to detect such free-floating litter,^40^ as shown in Figure 2. Additionally, both models used the same training set, with all three classes. Training three separate models, one for WHs, one for free-floating plastics, and one for entangled plastics may improve overall performance of detection of each class. This approach could also enhance the transferability across river systems, as the results suggest that only the free-floating plastic model would benefit greatly from retraining. However, using multiple separate models will make the monitoring process more complex.

### WH coverage decreases in downstream direction

#### WH pixel classification from Sentinel-2

**Figure 3:**
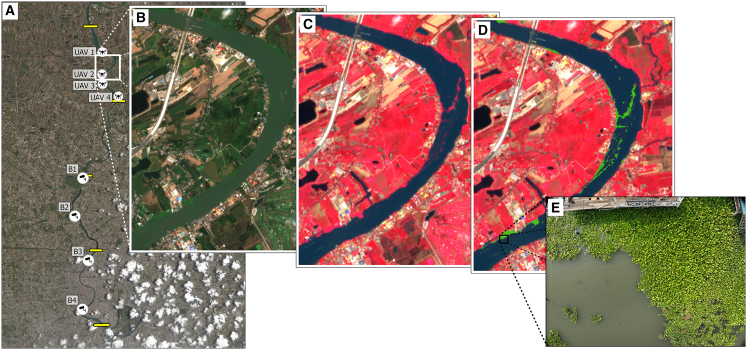
Example of the water hyacinths pixel classification at a certain location within the entire river extent (A) Reference map indicating the location of the example within the study area. (B) True-color Sentinel-2 imagery acquired on March 16, 2025. (C) The same image displayed in false-color composite of the water column prior to classification. (D) The same image but with water hyacinth pixels classified and highlighted in green. (E) UAV image taken at location UAV 2 on March 20. This example of a WH patch retained between building structures is used as no Sentinel-2 imagery with suitable cloud conditions was available on the same date as *in situ* imagery at any location.

The application of the Sentinel-2 WH detection algorithm classifies pixels at a spatial resolution of 10 × 10 m. Figure 3 shows the classification for a partial Sentinel-2 scene acquired on March 16, 2025. Figure S1 shows the spectral signatures and the difference between WHs and open water from hand-annotated 10 × 10 m Sentinel-2 pixels (*n* = 600) from the Saigon River, as reported by Janssens et al.^26^

### Spatial variation

**Figure 4:**
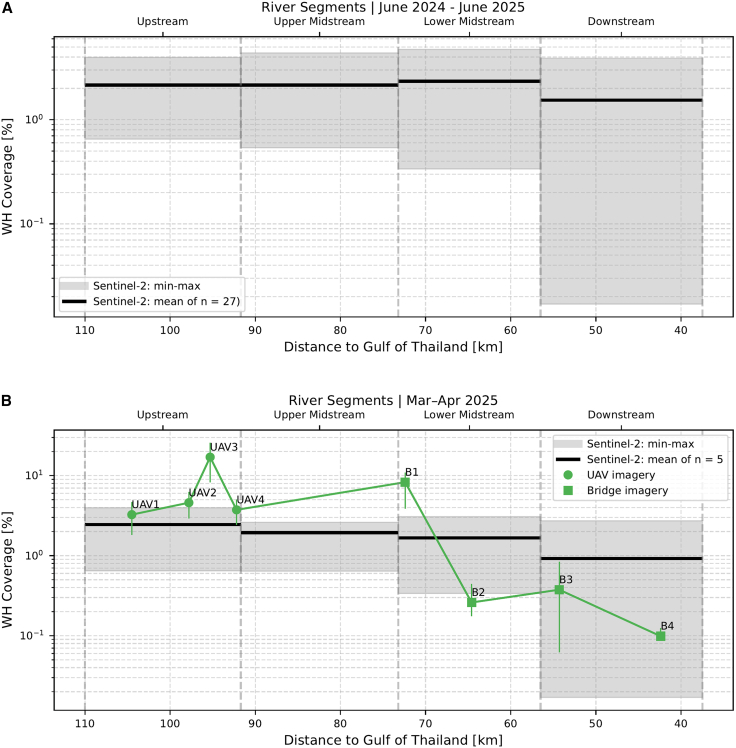
WH coverage along river segments (A) June 2024-June 2025 retrieved with Sentinel-2. (B) March-April 2025 with UAV and bridge imagery, compared to Sentinel-2 results. Data are represented as mean with minimum and maximum as error bars.

The mean classified WH area differed significantly among distance categories between June 2024 and 2025, as indicated by a Kruskal-Wallis test (*χ*^2^ = 20.98, df = 3, *p* = 0.0001). The annual average WH coverage, shown in Figure 4A, was 2.05%, ranging between 2.34% lower midstream and 1.55% downstream. A decreasing trend of WH coverage toward the river mouth is supported by a positive Spearman correlation; however, the significance of the correlation is weak (*ρ* = 0.16, *p* = 0.1035, *n* = 108). Throughout the study period, Sentinel-2 detects an average WH coverage of 1.81% across the study period, decreasing from average 2.44% upstream to 0.92% downstream, shown in Figure 4B. This declining spatial trend (Spearman, *ρ* = 0.46, *p* = 0.0425, *n* = 20) is consistent with findings from the Saigon River.^26^ Object detection during the same period showed a similar trend, although not significant (*ρ* = 0.62, *p* = 0.1017, *n* = 8). Compared to Sentinel-2, object detection revealed a higher WH coverage upstream by a factor of 1.3 (UAV1) to 7 (UAV3), and lower coverage downstream by a factor of 0.1 (B1) to 0.4 (B3).

**Figure 5:**
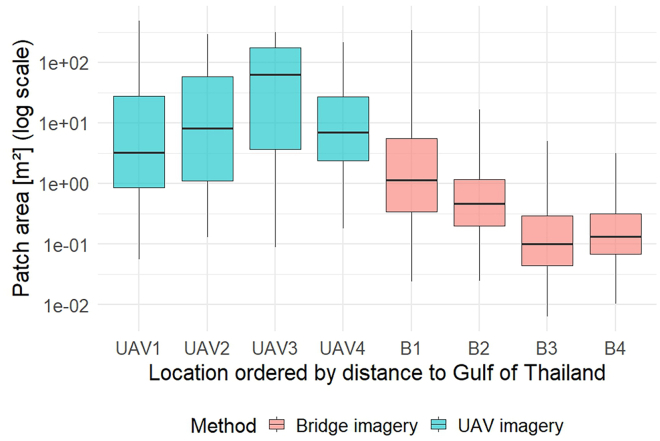
Mean patch sizes are up to a factor 162 larger upstream (UAV3) than downstream (B3) Maximum patch size is limited by the camera FOV. Data are represented as boxplots with the minimum and maximum as error bars.

WH coverage and mean patch sizes (m^2^) are lower downstream (Figure 5), likely due to disruption of mats by increased boat traffic and riverine infrastructure.^23^ This pattern further reflects the higher and cumulative urbanization along the lower Chao Phraya, both within and outside the Bangkok Metropolitan Region, compared to upstream areas.^41^ Additionally, stronger flow velocities or greater tidal forces may lead to fragmentation.^42^ These fragmented, but often dense clusters of small patches downstream may still fully occupy 10 × 10 m pixels in Sentinel-2 imagery, possibly explaining the higher values observed in Sentinel-2 data compared to bridge-based estimates.^26^ However, to validate this, the density of smaller patches (#/m^2^) should be investigated, as well as the minimum hyacinth coverage required for Sentinel-2 to classify a pixel as WH. UAV and bridge imagery can here validate and mitigate the resolution issues of Sentinel-2. On the other hand, the differences between Sentinel-2 and object detection are likely influenced by differences in spatial coverage and the degree of extrapolation required. Bridge and UAV imagery capture only a limited portion of the river, missing the broader variability visible in Sentinel-2 scenes, which can lead to mismatches in estimated coverage.

### Seasonal variation

**Figure 6:**
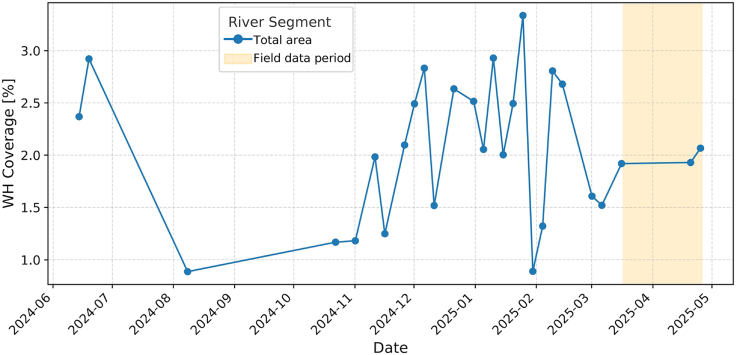
During the dry season, WH coverage was higher than during the hot and rainy seasons Between June and December 2024, only five images were available compared to 22 between December 2024 and June 2025.

Figure 6 shows a higher WH coverage (%) in the dry season, between November and February. This is similar to seasonal patterns observed in the Saigon River.^26^ Both regions show seasonality in the dry season, with low cloud cover. However, cloud cover poses a great uncertainty in the actual seasonality trend. In the Chao Phraya, only five images are available between July and December 2024. Additionally, WH coverage is not consistently highest during the dry season across all regions.^27^ Moreover, WH coverage over the spatial extent can be influenced by river obstructions, introducing variations that are independent of seasonal patterns, as is the case for the Chao Phraya.^43^ Therefore, the use of Sentinel-1 Synthetic Aperture Radar (SAR) data should be considered to map the spatial and temporal variation of WHs.^12^

### Plastic concentration in hyacinths increases in downstream direction

**Figure 7:**
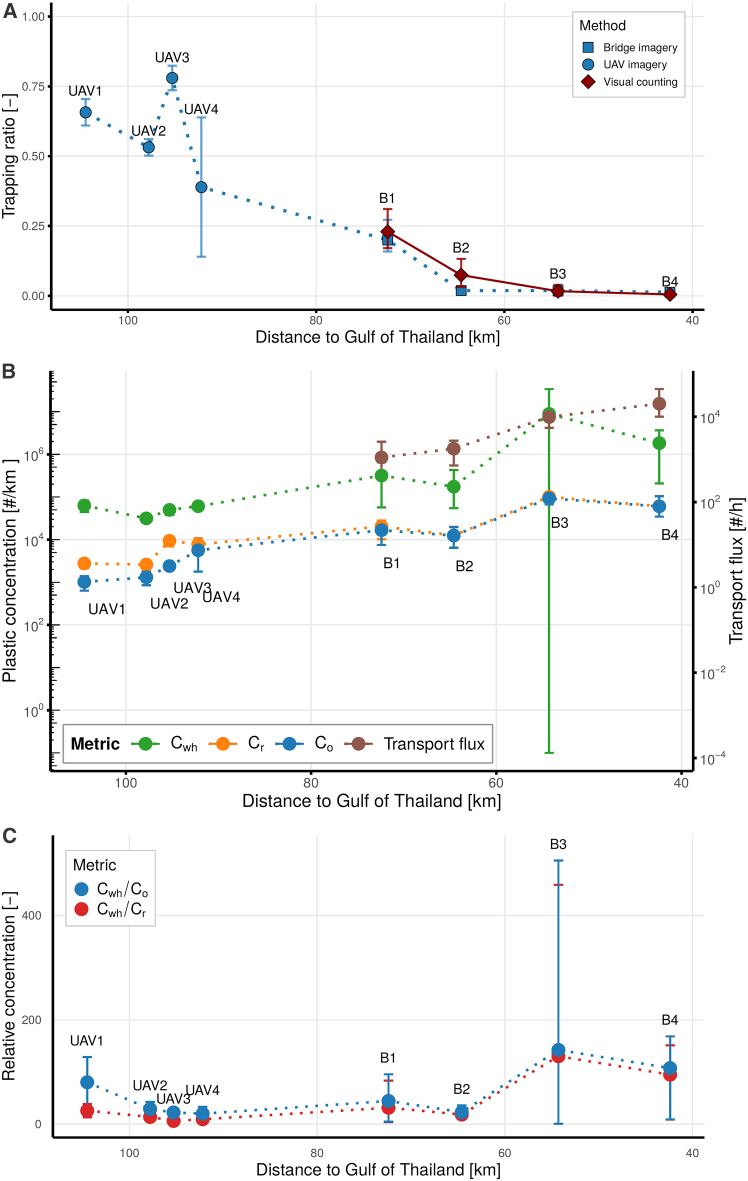
Spatial distribution of plastic trapping and concentration metrics toward the Gulf of Thailand (A) Trapping ratio, (B) plastic concentration in water hyacinths (*C*_wh_), in open water (*C*_o_), and of the total river surface (*C*_r_) are shown on the first *y* axis, with plastic flux on the second *y* axis. The global average of *C*_wh_ is 59 times higher than that of *C*_o_. (C) Relative concentrations of plastics: *C*_wh_/*C*_o_ and *C*_wh_/*C*_r_. Data are represented as mean with minimum and maximum as error bars.

Figure 7A shows that the trapping of plastics inside WHs decreases toward the Gulf of Thailand (Spearman, *ρ* = 0.93, *p* = 0.0022, *n* = 8). Averaged over all locations and periods, 32% of all plastics were found within WHs, ranging from 78% (UAV3) to 1.3% (B4). Only at the three most upstream locations, hyacinths carried the majority of floating plastics, with trapping ratios of 66% (UAV1), 53% (UAV2), and the highest location-specific mean of 78% at UAV3. It is still unclear what explains the lower trapping ratios downstream segments. The plastic flux still increases in the downstream direction, suggesting that there may be simply more plastics than the WHs can carry (plastic saturation), dependent on the season and influenced by higher and cumulative urbanization along the river downstream,^41^ with higher amount of plastics and the disruption of WH patches. Visual counting and bridge imagery shows similar trapping ratios across B1-B4, with small point-wise differences (−0.08 to 0.05). This consistency reinforces the validity of the results and improves transferability of using one or both of these methods. A reducing trapping ratio toward the ocean was also found in the Saigon, although with a much higher average trapping ratio of 73%.^24^ This emphasizes the variability of the capacity of WHs in plastic transport along both the spatial extent and among river systems.

In contrast to a negative trend in trapping ratio toward the sea, there is an increasing trend downstream of total number of entangled items per km^2^ WHs *C*_wh_ (#/km^2^) (Spearman, *ρ* = −0.81, *p* = 0.0218, *n* = (8) (Figure 7B). *C*_wh_ averages at 1.4 ⋅ 10^6^, and ranges from 3.1 ⋅ 10^4^ (UAV2) to 8.8 ⋅ 10^6^ (B3). Correspondingly, total river surface plastic concentration *C*_*r*_ and open water surface plastic concentration *C*_*o*_ (#/km^2^) both increase downstream, with Spearman correlations of *ρ* = −0.91 (*p* = 0.0046) and *ρ* = −0.95 (*p* = 0.0011), respectively. Plastic fluxes show a similar pattern, increasing from 1,114 (B1) to 20,025 (B4) items per hour, which is both smaller and larger than previously reported by van Calcar and van Emmerik,^31^ who observed fluxes between 2,460 and 5,340 items per hour 20–30 km from the river mouth.

The relative contribution of plastic concentration in WHs (*C*_wh_) compared to open water (*C*_*o*_) varies significantly, ranging from 20 times higher at location UAV4 to 142 times higher at location B3 (Figure 7C). The highest ratios are observed at the upstream and downstream locations, with plastic concentrations in hyacinths being 80 times higher than in open water at UAV1, 142 times higher at B3 and 107 times higher at B4. This indicates that WHs in these areas act as strong plastic accumulation zones.

Previously, the increase in *C*_wh_ downstream was associated with reduced hyacinth coverage and a slight drop in trapping ratio, *C*_*r*_ and *C*_*o*_ for the Saigon River.^25^ This does not hold for the Chao Phraya, where trapping ratios drop sharply from 73% to 1.3% and *C*_*r*_ and *C*_*o*_ both increase. At the same time WH coverage is low, and *C*_wh_/*C*_*o*_ is high downstream. This suggests that the sparse hyacinths accumulate high densities of plastic, arguably due to higher exposure to the overall plastic concentration in the river (*C*_*r*_) downstream; however, further research on the trapping mechanisms is needed. Therefore, it could be assumed that the trapping ratio is the driver of high *C*_wh_/*C*_*o*_ values upstream, while downstream WHs and plastics may mainly co-occur due to a similar response to the river flow dynamics, resulting in joint concentration.

### Physical sampling shows underestimation of entangled plastics

#### Plastic concentration by physical sampling

Physical sampling found approximately 74 plastic items per m^2^, compared to 5.3 items per m^2^ from bridge imagery within the same area as physical sampling (Table 2). The estimated hyacinth area (m^2^) and retrieved hyacinth mass (kg) suggest a wet biomass of approximately 8 kg/m ^2^ in the study area, comparable to the 10 kg/m ^2^ reported by Reddy and Sutton^44^ for low-density conditions. Sampling occurred in downstream locations with sparse, fragmented patches, consistent with such low-density hyacinth distributions. The higher concentration of plastics shown in physical sampling is in line with those in the Saigon River.^23^ This supports the idea that hyacinth’s traits, such as the extensive root structures, contribute to plastic trapping mechanisms. Additionally, bridge imagery likely missed plastics, as it usually fails to detect submerged items and transparent litter, and the detection of small plastics often fails.^40^ On the contrary, physical sampling included submerged, transparent and small plastics (≥0.5 cm), explaining the difference in items per m^2^ between bridge imagery and physical sampling.

#### Distribution of plastic types

**Figure 8:**
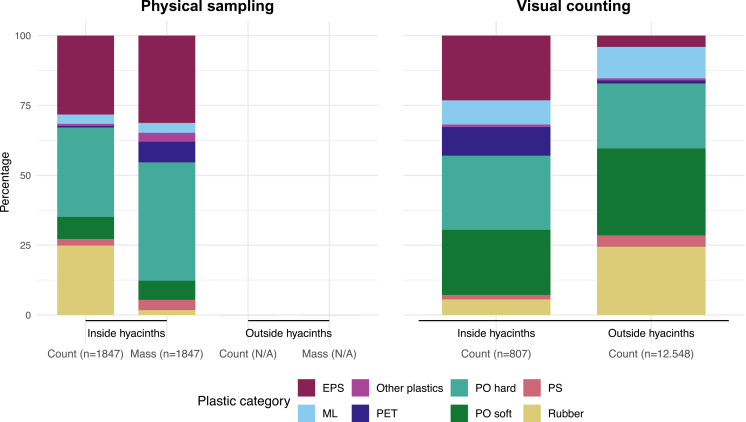
Physical sampling yielded both count and mass information of 8 distinct plastic categories This figure shows how it relates to the categorized count data retrieved with visual counting. Physical sampling solely focuses on plastics inside water hyacinths, whereas visual categorization includes counting data both in and outside water hyacinths.

Figure 8 shows the composition of plastic types, as retrieved from physical sampling and visual counting. Visual counting indicates a higher proportion of expanded polystyrene (EPS) and polyethylene terephthalate (PET) inside WHs compared to outside, with 23% vs. 4% for EPS and 10% vs. 1% for PET, respectively. This is similar to ratios found in Schreyers et al.^28^ Soft polyolefin (PO soft) and hard polyolefin (PO hard) display similar and predominant proportions both inside and outside hyacinths. Rubbers are found in a considerably higher portion outside hyacinths during visual counting (6% compared to 24%). However, physical sampling shows rubbers accounted for 25%. This could be attributed to the entanglement-prone structure of rubbers, easily getting trapped in hyacinth roots (Figure S3). Physical sampling shows a higher proportion of PO hard, and a lower proportion of PO soft and PET compared to visual counting. These differences could be influenced by the location where physical sampling was performed, given the plastic type concentrations found with visual counting (Figure S4).

The mass distribution further shows a higher proportion of PET, likely due to its large size class and generally unfragmented state.^23^ Although the count of rubbers is high, their mass contribution remains low, as the category mainly consists of small, lightweight, and elastic bands. Other plastics mostly consisted of lighters (full or empty), contributing more to mass. The mean mass per plastic type can be found in Table S4.

Our plastic type categorization in the Chao Phraya may assist local and national policymakers in identifying the most problematic plastic types for targeted mitigation measures. However, this categorization could be further improved through laboratory-based identification and exploratory studies on the dominant plastic types used for plastic items in different regions.

## Discussion

One of the main objectives of this research was to test the transferability of plastic-WH interactions, and field-based and RS detection methods. Most scientific work on plastic-WH interactions has been done on the Saigon River, Vietnam. Numerous anecdotal reports are available on similar processes in other rivers, including the Rio Ozama (Dominican Republic^45^) and the Citarum (Indonesia^16^^,^^46^). Yet, our paper quantitatively describes and analyzes the trapping of plastic pollution by WHs in another river system, the Chao Phraya. The available methods for WHs detection from space,^26^ and protocols to quantify the river-scale trapping of plastic using cameras, drones, and field sampling,^23^ were directly applicable to this river system. We are therefore confident that our combined work on these rivers can be further transferred to other rivers around the world, although adaptations may be required due to local circumstances and limitations.

### Transferability of plastic-WH interactions

WHs function as macroplastic aggregators in multiple river systems around the world and this is therefore a transferable phenomenon. This research finds that WHs trap up to 78% of all floating riverine macroplastics, and 32% on average over a 62.1 km spatial extent along the Chao Phraya River, Thailand. It was found that the trapping ratio and WH coverage show a decreasing pattern toward the river mouth. At the same time, we found that the concentration of plastics inside hyacinths increases in this direction. This paper suggests that the high plastic concentrations upstream are related to the plastic trapping capability of hyacinths. At locations where hyacinths are less abundant, downstream in this case, this research suggests that plastics and hyacinths jointly concentrate and co-occur due to a similar response to river flow dynamics.

### Transferability of plastic-WH detection

Sentinel-2 shows both its strengths and weaknesses. The overall detection capability of the Naive-Bayes classifier is promising, showing trends comparable to earlier research and cross-validation within this study. The free availability and high resolution of the data are promising for scalability; however, cloud cover poses a serious limitation for mapping temporal and spatial fluctuations given the variability in WH seasonality and distribution.

Object detection functions as a transferable approach to investigate plastic-hyacinth interactions, although limited for the detection of free-floating plastics. Free-floating debris and regional river characteristics may be too variable for the model. Training specialized models specifically for free-floating plastics may improve transferability to other river systems.

### Outlook to future work

Based on the global overlap of highly polluted rivers and thriving habitats for WHs, we expect that plastic-WH interactions are relevant in many other systems around the world. We therefore suggest to further test the transferability of these interactions and detection methods in other rivers with different geomorphological, hydrological, and plastic pollution characteristics. Additionally, evaluating sampling methods across varying drivers and environmental conditions, such as plastic loads, human presence, and river complexity, would support their harmonization and improve their reliability across different sites.^47^^,^^48^ Although the Chao Phraya and Saigon River differ in many aspects, they are also similar in terms of width, discharge, tidal dynamics, climate, and connection to densely populated areas. Smaller rivers and urban channels may reach much higher WH coverage proportions, and it is still unresolved how this may affect the trapping of plastic pollution. Furthermore, the RS-based WH detection methods may work less accurate due to the limited spatial resolution of Sentinel-2. Additional satellite imagery with higher resolution may offer additional opportunities for monitoring WHs in such systems.^12^^,^^49^ The applicability to large rivers should also be investigated further, such as the Amazon, Mekong, or Nile. These river basins are known to contribute to global plastic pollution and to face challenges from WH growth.^50^^,^^51^^,^^52^ The exact processes that result in trapping of plastics in WHs remain unresolved. From anecdotal evidence, it has become clear that the high correlation between plastic pollution and WHs can be attributed to both the trapping of plastics on and within the plants, and the co-occurrence of plastics and plant material.^25^ More detailed experiments, including tracking experiments, time lapses, or lab experiments may shed additional light on what factors determine the trapping and co-occurrence of plastics and WHs in river systems. Similarly, the release mechanisms remain unknown, and it is unclear whether this is driven by disintegration of the plant, or escaping of the plastic items from the plant. The plastic characteristics may also play an important role here. In both the Chao Phraya and Saigon, large portions of EPS foam were found within the WHs. However, it is clear that the composition of plastic pollution varies considerably between rivers around the world. For example, in European rivers higher portions of soft plastics are found, which are also often found in riparian vegetation.^31^^,^^53^ The effect of plastic characteristics on the trapping rate in WHs remains unresolved. Future work may also extend to smaller plastics, including microplastics and nanoplastics (MNPs). Previous work has found that WHs can effectively take up MNPs,^54^^,^^55^ it remains however unclear if WHs concentrate MNPs as much as floating macroplastics.

This paper evaluates the plastic-trapping role of WHs from large and small scale using satellite RS, drones, camera imagery, object detection, and extensive in-field measurements. Additionally, it expands investigation of plastic-hyacinth interactions to a different geographical context, the Chao Phraya, and covers the longest spatial extent examined in this context to date. Ultimately, this study supports the use of WHs as a transferable proxy in hyacinth-infested and plastic-polluted rivers worldwide. The use of this proxy could eventually support the development of large-scale plastic pollution monitoring strategies and targeted clean-up efforts.

### Limitations of the study

While this paper provides valuable research about the transferability of plastic-hyacinth relationships and detection models, several limitations require acknowledgment. First of all, the elaborate data collection over a 62.1 km extent yielded images with broad variance in camera height. These differences in camera height influenced the image resolution and potentially the minimum detectable plastic item size. However, cross-validation with previous research and simultaneous data-collection methods support the time and space-averaged results in this study. Further research into the spatial distribution of the plastic sizes of both entangled and free-floating plastics could help quantify these limitations. Second, post-processing included cross-evaluation of true hyacinth coverage but was only performed on images where hyacinths were not detected, yet entangled plastics were. As a result, the results may under-represent total hyacinth coverage and could be biased toward scenes where entanglement occurred. This potential bias could be accounted for in the future by applying systematic post-processing after object detection or by using segmentation for more accurate hyacinth detection, as demonstrated on UAV images by Schreyers et al.^23^ Third, tests of model transferability were limited to comparisons of model evaluation output, for object detection, and evaluation based on direct results for Sentinel-2. It is suggested that future research dives deeper into the generalization capacity of the models by, for example, testing the performance again after adding a small proportion of Chao Phraya training data to the training set, similar to what was done by van Lieshout et al.^56^ This would help assess how much additional data from another river system can improve model performance and transferability.

## Resource availability

### Lead contact

Requests for further information and resources should be directed to and will be fulfilled by the lead contact, Tim H. M. van Emmerik (tim.vanemmerik@wur.nl).

### Materials availability

This study did not generate new materials.

### Data and code availability

• The Sentinel-2 image data used in this work are part of the Copernicus Sentinel program and are freely available through the Copernicus Data Space Ecosystem. The access and processing of the data were performed through Sentinel Hub (Enterprise account), which was sponsored by the ESA Network of Resources (NoR). All UAV and GoPro imagery is publicly available at https://doi.org/10.4121/f771da08-8143-4a4b-9ad6-c8e711812a63.v1, just like the object detection results, annotations, physical sampling, and visual counting data.
• All original code has been deposited at 4TU and is publicly available at https://doi.org/10.4121/f771da08-8143-4a4b-9ad6-c8e711812a63.v1. The WHs classification tool using Sentinel-2 is available at https://github.com/GielHagenbeek/Waterhyacinth_classification_Sentinel2. The original YOLOv8 code is available at https://github.com/TianlongJia/deep_plastic_YoloV8^57^
• Any additional information required to reanalyze the data reported in this paper is available from the lead contact upon request.

## Acknowledgments

We are very thankful to Thanaphol Boodchuang (Asian Institute of Technology) who assisted with the fieldwork as drone pilot. We also acknowledge Chollada Phumsaard together with the municipal staff of the Bangkok Metropolitan Administration (Yellow Boat) for their contributions to the physical sampling campaign. Further thanks goes to Kathawut Prasarnnin and Nadhira Sagita Putri (The Ocean Cleanup) for the introduction and coordination with the Interceptor 019 team in Bangkok. Special thanks to Parattakorn Areerungruang and Makkatin Praphan from Miles Up Run Club for the warm welcome in Bangkok. Additionally, we thank everyone who helped and showed support throughout the process, especially Bianka Fábryová, Conor Murphy, Niek Westerink, Judith Hagenbeek, and Willem Hagenbeek. This work was supported by the ESA Network of Resources (10.13039/100014513NoR) sponsorship (5210uI). Finally, we thank the reviewers and editor whose comments helped further improving this manuscript.

## Author contributions

Conceptualization, G.W.A.H., T.H.M.v.E., and M.R.; data curation, formal analysis, investigation, validation, and visualization, G.W.A.H.; funding acquisition, G.W.A.H. and M.R.; methodology, G.W.A.H., T.J., and R.T.; project administration, G.W.A.H., K.B., P.K., T.H.M.v.E., and M.R.; resources, G.W.A.H., K.B., P.K., T.J.; software, G.W.A.H., M.R., and T.J.; supervision, T.H.M.v.E. and M.R.; writing – original draft, G.W.A.H. and T.v.E.; writing – review and editing, all authors.

## Declaration of interests

T.M. is employed by The Ocean Cleanup, a non-profit organization aimed at advancing scientific understanding and developing solutions to rid the oceans of plastic.

## STAR★Methods

### Key resources table

**Table:** 

REAGENT or RESOURCE	SOURCE	IDENTIFIER
**Deposited data**		

UAV imagery	This study	https://doi.org/10.4121/f771da08-8143-4a4b-9ad6-c8e711812a63.v1
Bridge-mounted camera imagery	This study	https://doi.org/10.4121/f771da08-8143-4a4b-9ad6-c8e711812a63.v1
Visual counting	This study	https://doi.org/10.4121/f771da08-8143-4a4b-9ad6-c8e711812a63.v1
Physical sampling	This study	https://doi.org/10.4121/f771da08-8143-4a4b-9ad6-c8e711812a63.v1
Ground truth annotations	This study	https://doi.org/10.4121/f771da08-8143-4a4b-9ad6-c8e711812a63.v1

**Software and algorithms**		

Python v3.8.10	Python Software Foundation	https://www.python.org
scikit-learn	Python Software Foundation	https://scikit-learn.org/
eo-learn	eo research team	https://eo-learn.readthedocs.io/
YOLOv8	Ultralytics	https://github.com/ultralytics/ultralytics
YOLOv8 models	Ultralytics	https://github.com/TianlongJia/deep_plastic_YoloV8
LabelStudio	Tkachenko et al., 2020	https://labelstud.io/
ExifTool	Harvey, 2016	https://exiftool.org/
Water hyacinth classification tool (Sentinel-2)	This study	https://github.com/GielHagenbeek/Waterhyacinth_classification_Sentinel2
Google Colab	Google	https://colab.research.google.com/

**Other**		

Sentinel-2 imagery	Copernicus Program	https://sentinels.copernicus.eu/

### Method details

#### Satellite remote sensing data

Sentinel-2 imagery offers high-resolution multispectral data (10–20 m depending on the band), covering red-edge, near-infrared (NIR), and shortwave infrared (SWIR) bands, which are well-suited for detecting floating vegetation due to their distinct spectral signatures.^26^ Atmospherically Sen2Cor^58^-corrected 12-band Sentinel-2 L2A scenes were retrieved from SentinelHub, covering the entire study area from 1 June 2024, to 1 June 2025, including a 5 km buffer at both the northern and southern river extents. A total of eight tiles were used to retrieve the scenes, equally divided across the four river segments shown in Figure 1A. Scenes with cloud coverage above 80% were not used. Only dates with scenes available across all river segments were used, resulting in 27 scenes per segment for full-year analysis and 5 scenes per segment aligned with the fieldwork period.

To estimate WH coverage, we used a Naive Bayes–based classifier, which was trained on hand-annotated 10 × 10 m Sentinel-2 pixels (*n* = 600) from the Saigon River using the *scikit-learn*^59^ Python package with an 80:20 training–testing split by Janssens et al.^26^ Training and test sets were based on visual selection of reflectance values of Sentinel-2 bands, Normalized Difference Vegetation Index (NDVI) and Floating Algae Index (FAI). Figure S1 illustrates the spectral reflectance and index-based differences between WHs and open water derived from this dataset, including NDVI and FAI values, demonstrating the separability used to train the classifier. The model was implemented in Google Colab using the *eo-learn*^60^ collection of Python packages. We applied the model as provided, without retraining on new data from the Chao Phraya River.

The classifier inputs 5 features, i.e., NDVI, FAI,^61^ and the blue (B2), green (B3) and short-wave infrared bands (B12) and estimates the likelihood of hyacinth presence. After cloud masking using Python package *S2cloudless*,^62^ the remaining pixels were categorized by the built-in Scene Classification Layer (SCL) with water (SCL = 6), bare soil (SCL = 5), or vegetation (SCL = 4). A total of 2006 ha of river surface over 79 km of river was used, divided into four segments to align with the distribution of *in-situ* data collection: upstream (678 ha), upper midstream (405 ha), lower midstream (451 ha), and downstream (472 ha) (Figure 1A). The output of the model yields the coverage of WHs as percentage of the river (section) area.

#### Field data collection

Measurements were performed during the dry season, between 17 March and 26 April 2025. Four data collection methods were used: (1) the collection of bridge-mounted camera imagery, (2) UAV imagery, (3) visual counting, and (4) physical sampling of WH patches. All measurements were spread over a 62.1 km length of the river, with the most northern part 104.5 km upstream of the Gulf of Thailand and the most southern part 42.4 km away (Figure 1). Distance to the Gulf of Thailand was measured along the river centerline, following the channel. The meander cutoff under location B1 was included in the total distance, whereas the longer meander loop was not.

#### Bridge-mounted camera imagery

Four bridge locations were revisited during four periods, during which a single GoPro Hero 11 (GoPro, Inc., San Mateo, USA) was deployed on one bridge per day in a fixed order (B1–B4). At B1 the GoPro was deployed on the South side to avoid capturing a ferry and on B2-B4 on the North side. The GoPro was mounted on a 1 m arm to capture the water surface at nadir without the bridge structure in frame (Figure 1B). Images were taken using the 8:7 linear lens. Per bridge, five waypoints were equally distributed across the width of the river, to capture characteristics over the full width of the river. Waypoint spacing prevented image overlap at all bridges. The Field of View (FOV) at the outer waypoints was set to cover the river surface only, avoiding the riverbank and overhanging structures, such as piers or buildings. All waypoints were revisited in six rounds per date, evenly split between morning and afternoon, except B3 in period three. Each round proceeded west to east. The GoPro was set to take an image every 10 s during a span of 5 min per waypoint, planned to yield 31 images per round per waypoint. The final amount of images captured per waypoint and round was influenced by battery level, camera-overheating and manual mistakes. In total 14,742 images were taken and used without further filtering. Camera height at waypoints 1 and 5 was measured using an MK202 distance laser reflecting off the riverbank at water level, as direct measurements from the water surface were not possible due to low reflectivity. Distances were recorded once at high tide and once at low tide. Bridge center height was estimated from the bridge structure elevation, and for waypoints 2 and 4 the average of the outer and middle waypoints was used. Camera height of each individual image was taken into account, and used as input for the calculation of the ground sampling distance (GSD), which we elaborate on in the ‘imagery-processing’ section.

#### UAV imagery

On 20 March and 4 April, UAV images were collected at four river cross-sections, at locations different from the bridge-mounted imagery, as shown in Figure 1A. Each location was revisited in the morning and afternoon for equal distribution between tides (except for UAV4, on the 20th of march, which was only visited in the afternoon). A DJI Mavic 3 Enterprise (SZ DJI Technology Co., Ltd., Shenzhen, China) with RGB camera, a 4/3 CMOS sensor and 20 megapixels was used. UAV flights were performed by a parallel flying pattern,^63^ crossing the river perpendicularly to the water flow, as shown in Figure 1C. Per location, the UAV crossed the river between 38 and 70 times spread over two periods. The number of river crossings per location varied due to battery levels, as time did not allow for recharging after every flight. Flight height and river width influenced the time required per crossing, further constraining consistency. Flight height was aimed to average 10 m for optimal performance,^64^ with deviations caused by surrounding conditions at the take-off location. To limit capturing the same flowing hyacinths, each returning river crossing moved slightly further against the direction of the river flow. Parallels were not aimed to be overlapping. Images were taken at a constant interval while flying from riverbank to riverbank. A total of 9,884 images were taken. Images of the riverbank were filtered out manually (*n* = 3,482). All other UAV images were used (*n* = 6,402).

**Table:** Details per bridge and UAV locations, including rounds of observations per period.

Location ID	Lat	Lon	km upstream	River width [m]	Rounds per period			
–	–	–	–	–	17–21 Mar	24–28 Mar	8–12 Apr	22–26 Apr
UAV 1	14.12086	100.5265	104.5	300	33	–	37	–
UAV 2	14.08607	100.5254	97.8	390	23	–	15	–
UAV 3	14.06883	100.5274	95.3	227	37	–	27	–
UAV 4	14.04866	100.5533	92.2	247	10	–	26	–
B1	13.91570	100.4939	72.4	191	6	6	6	6
B2	13.85368	100.4803	64.6	280	6	6	6	6
B3	13.78107	100.5024	54.3	333	6	6	7	6
B4	13.70090	100.4921	42.4	375	6	6	6	6

**Table:** Camera setup and image statistics for each location

Location ID	Camera height [m]			GSD [cm/pixel]		FOV [m^2^]		# Images after filtering	Annotated Images	Annotated Items
	min	max	avg	Min	max	min	max	–	–	–
UAV 1	4.59	19.18	10.82	0.12	0.51	31	545	2246	9	104
UAV 2	9.26	14.39	11.16	0.25	0.38	127	307	1592	7	242
UAV 3	10.03	17.62	11.73	0.27	0.47	149	460	1688	10	47
UAV 4	9.46	12.83	11.11	0.25	0.34	133	244	876	9	136
UAV total	–	–	–	–	–	–	–	6402	35	529
B1	11.10	12.00	11.46	0.46	0.50	574	671	3874	17	426
B2	10.40	12.40	11.20	0.43	0.51	114	717	3592	10	50
B3	5.50	6.50	5.90	0.23	0.27	132	197	3614	10	71
B4	6.20	10.70	8.00	0.26	0.44	156	534	3662	10	339
Bridge total	–	–	–	–	–	–	–	14742	47	886
Total	–	–	–	–	–	–	–	21144	82	1415

#### Visual counting of floating plastic

Visual counting was performed at each waypoint, nearly simultaneously with bridge imagery. Over the span of 5 min, all litter flowing into the visual FOV was counted. Debris already present in the FOV before counting began, whether stationary or exiting, was not counted. The visual FOV is different from the bridge-mounted camera FOV, with a constant width estimated at 15 m. When plastic flow was too fast to count (>27,000 items h^−1^), the number was estimated by groups of 10 or counting durations was reduced to less than 5 min (*n* = 26). The minutes of counting was noted per observation. For each counted item it was noted whether it was free-floating or entangled in WHs (Figure 1C). Additionally, all litter was assigned to its corresponding plastic category (Table S1), based on the common plastic type classifications of the item, as seen from the bridge.^28^^,^^31^ We used 8 distinct groups for plastic categorization: polyethylene terephthalate (PET), soft polyolefin (POsoft), hard polyolefin (POhard), multilayer plastics (ML), polystyrene (PS), expanded polystyrene (EPS), other plastics, rubbers and other litter. The categories POhard and POsoft encompass both polyethylene (PE) and polypropylene (PP). During high plastic flow events, categorization took place for 1 min following an extrapolation to the total plastics counted in the total counted minutes. A total of 13,491 items were classified, of which 13,355 being polymers and rubbers. Other litter (*n* = (136) is not further taken into account in the analysis. From here on, plastics will refer to both polymers and rubbers. From the visual counting observations, the surface plastic transport rate [#/h] was estimated following Schreyers et al.,^24^ by normalizing plastic counts by observation time and scaling by river width. Trapping ratio [-] was retrieved by dividing the summed counted entangled plastics by the summed total plastics per waypoint per round. Hereafter, the trapping ratio was averaged across waypoints and rounds to get a representative value for the river width per date and location.

#### Physical sampling

Physical sampling of WH patches was conducted over four consecutive days, from 1 to 4 April, between 08:30 and 15:00 (UTC+(7) each day. The sampling took place working together with the river cleaning team of the Bangkok Metropolitan Administration (BMA) and within their perimeter, the Bangkok Metropolitan Area, as outlined in Figure 1A. A team of 2–3 people collected WH patches from a boat using round dip nets, which are ordinarily used by the BMA for litter collection, as shown in Figure 1E. The nets were submerged directly in front of the patches to minimize the simultaneous collection of entangled and free-floating debris. After extraction, all caught material was placed into a basket, after which WH was separated from anthropogenic debris. The wet mass per WH patch was measured on board using a scale. In some cases (*n* = 8), multiple small patches were combined and weighed together, and the anthropogenic debris was sorted collectively. On board, all plastics were sorted and counted according to the categories described in the Visual Counting section. Only plastics ≥0.5 cm in any dimension were included.

All collected plastics were preserved for subsequent mass analysis on shore. A representative quarter of the total number of items was then selected, categorized, and weighed to calculate the mean mass per plastic type (kg). Plastic concentrations per WH were recorded as (1) plastic area concentration [# items/m^2^], and plastic mass concentration [g/kg]. In total, 58 patches were sampled, together making up for approximately 200 kg of wet biomass. A total of 1,832 plastic items were found, making up for 5 kg in total. The estimated surface area of individual samples ranged from 0.03 to 2.25 m^2^, with an average of 0.57 m^2^, totaling 25 m^2^ across all sampled WHs.

#### Imagery processing

This section describes the imagery processing using the object detection model applied to bridge-mounted camera and UAV imagery.

#### Model description

YOLOv8 deep learning architecture^33^ was used to detect WHs and plastics in all UAV and bridge imagery. YOLOv8 is a one-stage convolutional neural network for real-time object detection. It has two main parts: a backbone that extracts multi-scale features by balancing depth, width, and resolution, and a detection head that detects objects and outputs bounding boxes to show the object type and location.

Two models developed on Saigon River imagery were applied directly to the Chao Phraya River, without any retraining. Due to YOLOv8’s input size limitation (maximum 640 × 640 pixels), these models were originally developed using two separate imagery processing methods. The Hyacinth Model was trained on resized images (*n* = (218) and the Plastic Model was trained on tiled images (*n* = 11,408).^25^ Both models are trained on three classes: (1) WHs, (2) free-floating plastic, and (3) plastic entangled in WHs.^65^ The Plastic Model was used to retrieve both free-floating and entangled plastic counts from tiled images. The Hyacinth Model was used to retrieve WHs from the resized images. Tiling cut an original image into 56 (GoPro) or 48 (UAV) tiles. Resizing takes the original image and reduces it to image size 640 by 640 pixels. All UAV and bridge imagery was used as input for both models, after which all detections were matched back to the original image for integrated analysis.

#### Model performance evaluation

To evaluate the model performance on the Chao Phraya River a validation set of 82 images was created out of a total of 21,144 images (70% GoPro, 30% UAV). A total of 1,415 objects were annotated using LabelStudio^66^ (54% GoPro, 46% UAV), used for the HyacinthModel. Additionally, these annotations were cut into tiles to align with the tiled images, which may have excluded objects partially outside tile boundaries, resulting in 1,346 annotations for the PlasticModel. The number of annotated items per class was kept approximately equal (Table S2). For both models, the bounding box coordinates were recalculated to the original image size.

Model performance was evaluated on a hand-annotated validation set using commonly used YOLOv8 metrics^34^^,^^67^^,^^68^: (1) precision, the ratio of correct detections to total detections; (2) recall, the ratio of correct detections to actual objects; (3) mAP50, the mean average precision at an Intersection over Union (IoU) threshold of 0.50; and (4) mAP50–95, the mean average precision averaged across IoU thresholds from 0.50 to 0.95. The model performance evaluation was run with a default confidence threshold of 0.5.

#### Model output settings

To determine suitable confidence scores for running the two models, validation set predictions were evaluated against ground truth annotations. IoU was used to determine matches between predicted and ground truth bounding boxes. A detection was counted as a True Positive (TP) if the IoU was ≥0.5 and the predicted and ground truth class labels matched. Predictions without matching ground truth were counted as False Positives (FP), and unmatched ground truth boxes as False Negatives (FN). Predictions were evaluated at confidence levels ranging from 0.00 to 1 in increments of 0.05. The optimal confidence levels for each method were selected based on the highest F1-score, the harmonic mean of precision and recall. For the PlasticModel, the confidence thresholds determined for free-floating plastics were used. For GoPro entangled plastics, the applied threshold of 0.10 corresponded to an F1 of 0.47.

**Table:** Highest F1 values found per model and method and the corresponding confidence thresholds.

Model	Class	Method	F1_max_	Confidence threshold	Confidence threshold applied
HyacinthModel	Hyacinths	UAV	0.72	0.30	0.30
–	–	GoPro	0.61	0.20	0.20
PlasticModel	Free-floating plastics	UAV	0.36	0.45	0.45
–	–	GoPro	0.30	0.10	0.10
–	Entangled plastics	UAV	0.45	0.45	0.45
–	–	GoPro	0.61	0.30	0.10

#### Post-processing of object detection output

All FOVs and bounding boxes were recalculated from pixels to surface area in square meters [m^2^]. Recalculations were performed using the Ground Sampling Distance (GSD) [cm/pixels]:

$$GSD=\frac{S_{w}\times H}{F_{l}\times \omega_{i}}\text{,}$$

where *H* is the camera height, *S*_*w*_ is the camera sensor width, *F*_*l*_ the camera focal length [mm], and *ω*_*i*_ the image width in pixels.^69^ Except for height, all variables in this equation remained constant per UAV or GoPro. Height for UAV was retrieved using ExifTool^70^ and specified per image. For GoPro imagery, the in-field reported height per waypoint per bridge, averaged over tides, was used.

All WH bounding boxes were corrected by factor 0.79 to comply with the predominant ellipse shape of WH patches.^24^ All instances where entangled plastics but no WHs were identified were manually cross-checked (*n* = 164). Cases where the image was fully covered by WHs were corrected with the same area as the FOV (*n* = 138). Others with partial WH coverage were corrected by FOV multiplied with the ellipse factor (*n* = 11). For all other cases hyacinth area was kept 0 (*n* = 15). In cases where the WH coverage exceeded the FOV, (*n* = 202), the corrected area was calculated as FOV × 0.79, assuming that the overlaying bounding boxes resulted from multiple single WH patches.

Additionally, uncorrected bounding boxes of detected plastics (both free-floating and entangled) were grouped into 11 size classes from 0.01 to 0.1 up to 0.9–1.0 and above 1.0 m^2^. For both UAV and GoPro images, up to 10 images per class were used as input, and object detection was applied based on a 0.1 confidence level. All retrieved bounding boxes were examined, and the ground truth object type was noted. The majority of objects in size classes above 0.1 m^2^ were not plastics, and the majority of detected objects also fall within this class size (Figure S2). Plastic size or size filtering was not further applied or used for plastic–WH interaction metrics.

#### Plastic–water hyacinth interaction metrics

With the retrieved bounding boxes of WHs, counts of free-floating and entangled plastics, and FOVs, the following metrics are calculated: trapping ratio [-], water hyacinth surface coverage [m^2^], water hyacinth patch size [m^2^], the total river surface plastic concentration *C*_*r*_ [#/km^2^], the non-hyacinth-covered (open) surface plastic concentration *C*_*o*_ [#/km^2^], the concentration plastics inside hyacinths *C*_wh_ [#/km^2^], and consequently the ratios *C*_wh_/*C*_*r*_ and *C*_wh_/*C*_*o*_, which compare plastic concentration in hyacinths to the total river and open water concentrations, respectively. Calculations are shown in Table S3.

#### Statistical analysis

Normality of the data was assessed using the Shapiro–Wilk test. As normality was not found, differences between distance categories were evaluated using the non-parametric Kruskal–Wallis test. Relationships between relevant metrics and distance to the Gulf of Thailand were assessed using Spearman’s rank correlation, which is robust to non-normal distributions and captures correlation without assuming linearity. The correlation is based on the mean values per location. Statistical significance was defined at *p* < 0.05. The minimum and maximum values per location, over the complete field work period, are also calculated and included as error bars.

### Quantification and statistical analysis

Statistical details of experiments, including the number of observations (n) can be found in the figures, tables, Results and Methods sections. Spearman’s rank correlation was used to test relationships between relevant metrics. Correlations were calculated on mean values per location, with statistical significance defined at *p* < 0.05. Correlation coefficients (*r*) and associated *p*-values were also reported to assess linear relationships where applicable. No data points were excluded. All statistical parameters, including per-class F1-scores are reported alongside figures, tables, and in the Results section. No randomization or blinding was applied, as the study relied on deterministic classification and observational measurements.

## Supplemental information

Document S1. Figures S1–S4, Tables S1–S4, and Reference
