# Supplementary material for: Exploring transferability of plastic-water hyacinth interaction and detection in rivers
Source: iScience. 2026 Jun 4;29(6):116238. doi: 10.1016/j.isci.2026.116238 (PMC13266208; doi:10.1016/j.isci.2026.116238)
Supplement: Document S1. Figures S1–S4, Tables S1–S4, and Reference [file mmc1.pdf]

iScience, Volume *29*

Supplemental information

Exploring transferability of plastic-water hyacinth interaction and detection in rivers

Giel W.A. Hagenbeek, Tim H.M. van Emmerik, Tianlong Jia, Pummarin Khamdahsag, Kittiphon Boonma, Riccardo Taormina, Thomas Mani, and Marc Rußwurm

1. Spectral signals of water hyacinths and water surface

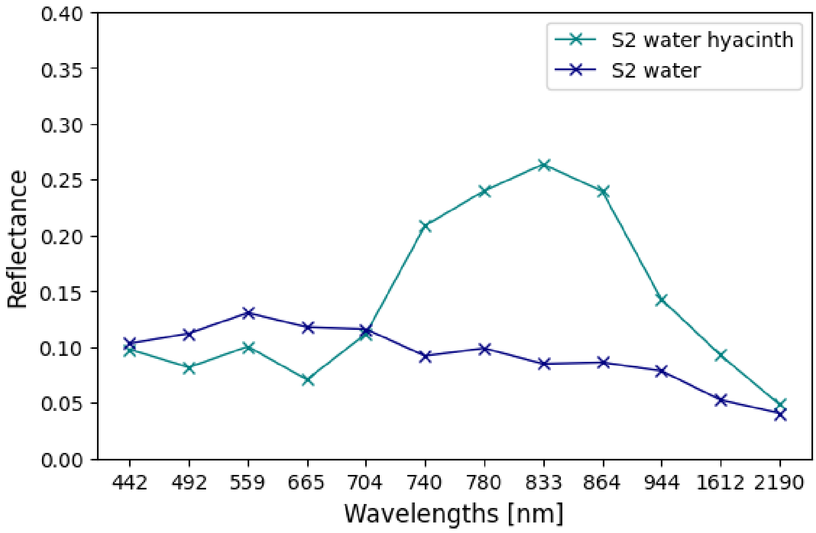

(a)

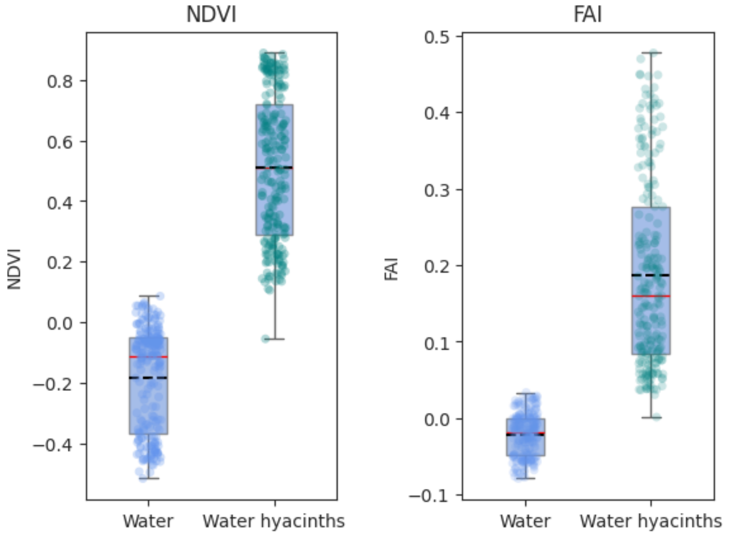

(b)

Figure S1: Differences in reflectance values and spectral signal for water and water hyacinths, based on hand-annotated 10 × 10 m Sentinel-2 pixels (n = 600) from the Saigon River by Janssens et al. [1]. (a) Distribution of reflectance values of water hyacinths and water across different wavelengths and (b) NDVI and FAI spectral signals of WHs and water. Data are represented as boxplots with minimum and maximum values as error bars, with the red line representing the median value and the black dashed line the mean.

2. Plastic Categories and Items

Table S1: Plastic categories with corresponding items.

Plastic category	Plastic items
PET	Water and soda bottles
	Salad bowls
PO hard	Transparent cups
	Transparent lids
	Packaging straps
	Bottle caps
	Straws
	Food baskets
	Foldable cutlery
	Milk bottles
	Shampoo bottles
	Yoghurt containers
PO soft	Bottle labels
	Decoration straps
	Plastic bags
	Single layer food wrappings
	Single layer transparent wrappings
PS	Cutlery non-foldable
	Cloth hangers
	Non-transparent coffee cup lids
	Non-transparent snackbar food containers
EPS	Foam fragments
	Food foam plates
	Packaging foams
	Isolation material
Multilayer	Multilayered wrappings or packaging
Other plastics	Strip of pills
	Ointment tubes
	Footwear
	Cigarettes
	Duct tape
	Lighters
	Fake leather
	Cable housing
	Filled garbage bags
	Fake flowers

3. Annotation

Table S2: The amount of annotated items for each model and class.

**Model**	**Class**	**No. annotated items per class**
	Water Hyacinth	485
HyacinthsModel	Free-floating plastics	632
(n=82)	Entangled plastics	298
	All classes	**1415**
	Water Hyacinth	457
PlasticModel	Free-floating plastics	600
(n=4288)	Entangled plastics	289
	All classes	**1346**

4. Size class and item distribution of detected plastics

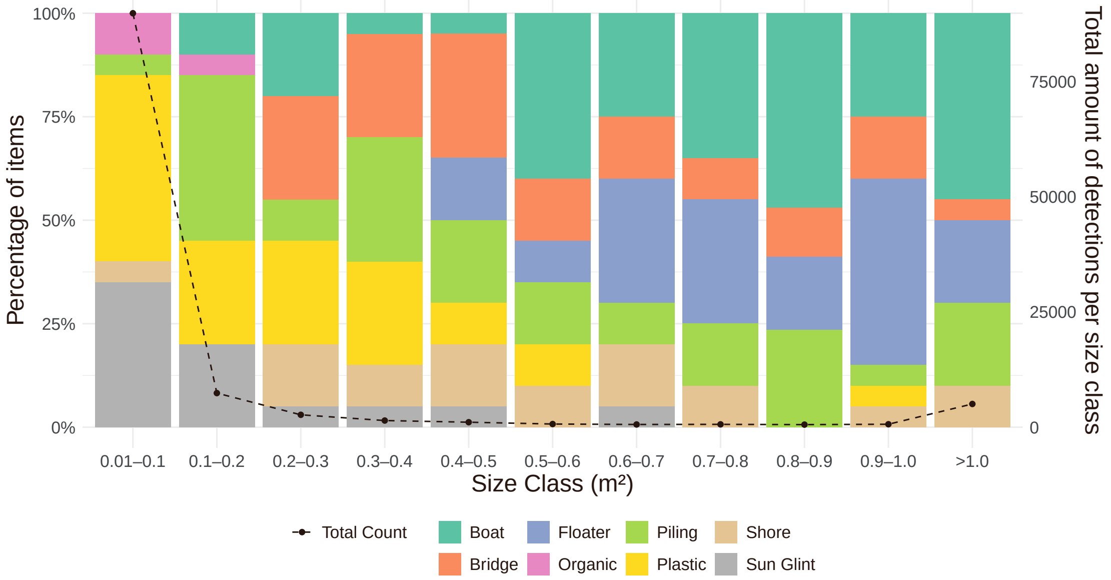

Figure S2: The histograms show the distribution of the ground-truthed item type, which object detection classified as plastic, categorized per size class. The black line show the total amount of object detected identified items in the corresponding size class.

5. Metric calculations

Table S3: Metric calculations used in the study, with descriptions and formulas.

**Metric**	**Description**	**Formula**
Trapping ratio	Fraction of detected plastics that are entangled in water hyacinths	$r_{\mathrm{ent}} = \frac{N_{\mathrm{ent}}}{N_{\mathrm{ent}} + N_{\mathrm{free}}}$
Water hyacinth surface coverage	Proportion of the water surface A_r (total FOV) occupied by water hyacinths A_{wh}	$f_{\mathrm{wh}} = \frac{A_{\mathrm{wh}}}{A_r}$
Total plastics concentration	Concentration of all plastics per unit river surface area	$C_r = \frac{N_{\mathrm{ent}} + N_{\mathrm{free}}}{A_r}$
Free plastics concentration	Concentration of free-floating plastics per unit open water area	$C_o = \frac{N_{\mathrm{free}}}{A_r - A_{\mathrm{wh}}}$
Entangled plastics concentration	Concentration of entangled plastics per unit water hyacinth-covered area	$C_{\mathrm{wh}} = \frac{N_{\mathrm{ent}}}{A_{\mathrm{wh}}}$
Symbols and Units		
N_{ent}	Number of entangled plastic items	
N_{free}	Number of free-floating plastic items	
A_r	Water surface area of the scene (FOV) [m^2]	
A_{wh}	Area covered by water hyacinths [m^2]	
C_r	Total plastics concentration [items/m^2]	
C_o	Free-floating plastics concentration [items/m^2]	
C_{wh}	Entangled plastics concentration [items/m^2]	
r_{ent}	Trapping ratio (%)	
f_{wh}	Water hyacinth surface coverage (%)	

6. Microplastics between water hyacinth roots

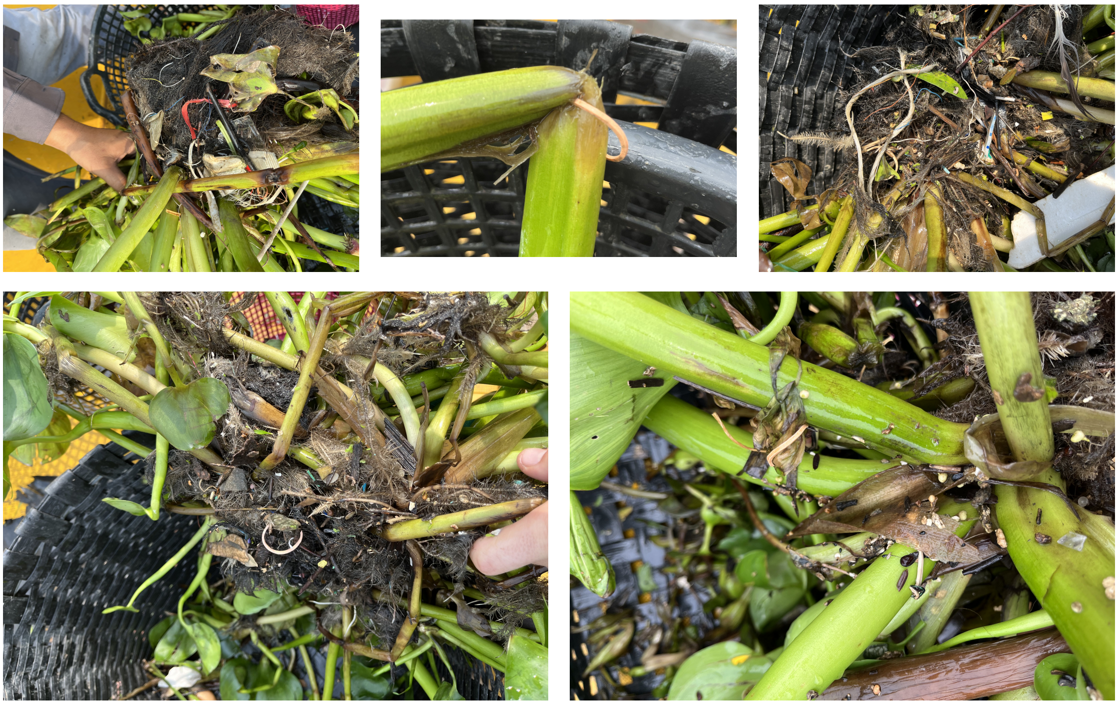

Figure S3: Water hyacinth root structures entangling and attracting rubbers, macro- and microplastics. Photos were taken during the physical sampling campaign (1–4 April, 2025)

7. Distribution of plastic category inside and outside hyacinths.

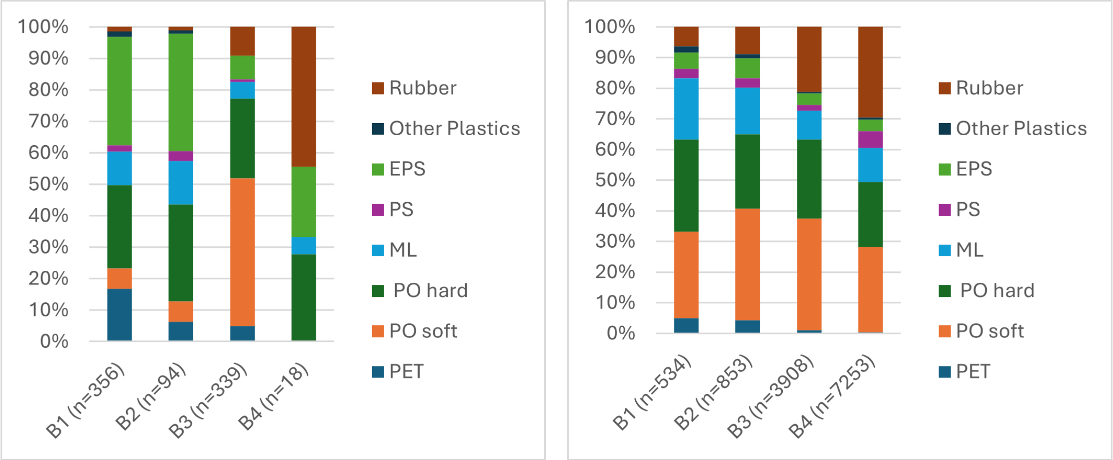

(a) (b)

Figure S4: Plastic category distribution inside and outside water hyacinths as seen from each bridge. (a) Distribution of entangled plastics per bridge. (b) Distribution of free-floating plastics per bridge.

8. Mean mass per plastic category

Category	PET	PO soft	PO hard	PS	ML	EPS	Other plastics	Rubber	Total
# Weighted items	10	56	309	25	40	200	10	245	895
Mean mass (g)	34	2.34	3.59	4.27	2.89	3	11.27	0.19	2.71
Total counted items	11	147	590	61	43	522	14	459	1847
Estimated total mass (g)	374	344.66	2119.42	260.35	124.38	1566	157.78	86.93	5033.51

Table S4: Mean mass per plastic category derived from physically sampled water hyacinths, used to estimate total plastic mass from item counts.

References

[1] Niels Janssens, Louise Schreyers, Lauren Biermann, Martine Van Der Ploeg, Thanh Khiet L. Bui, and Tim Van Emmerik. Rivers running green: Water hyacinth invasion monitored from space. *Environmental Research Letters*, 17, 2022. doi: 10.1088/1748-9326/ac52ca.
